# The Nude in Medical Journalism

**Published:** 1896-09

**Authors:** 


THE NUDE IN MEDICAL JOURNALISM, or nastiness gone mad. What possible connection can there be, or be imagined, between the picture of a naked woman wallowing on the grass and medical literature? I give it up. Several of our journals—one,—heretofore very decent—have somehow, a strong penchant for the nude, and each month regale (disgust) their readers with pictures that are nothing, if not wanton, bad—nasty; pictures of naked women. The St. Joseph *Medical Herald* went largely into the nude, as supposed embelishments to its pages of medical reading; the Kansas City imitation Red Back—Boteler's *North American Medical Review*, has been full of naked pictures; so notorious it became, in fact, that our junior, who is a most modest man—had to look out for it each month and hide it from our office boy for fear of corrupting his pure young mind.

But Great Scott! here comes a brand new departure—a whale of nastiness to the little scaly things that have gone before—*Moody's Medical Magazine* (see the alliteration; musical, but—) which outdoes its prurient predecessors above mentioned, in the nasty business, and utterly ruins an otherwise nice looking pamphlet—by the introduction on the title page of the picture of a naked woman—with an idiotic leer on her countenance—stretched full length on the grass in the most wanton and lascivious attitude imaginable. It is the coarsest, most revolting and utterly disgusting exhibition we ever saw. What do the publishers of such stuff take their readers for? Alas for Southern medical journalism! when it comes to this.

The publication just referred to—*M. M. M.*—was foreannounced by a flaming prospectus—flaming with the photo of the editors, and one of some contributor, with colored inks and trimmings to match—and we looked with a sort of longing for the thing itself. By and by—an esteemed correspondent *sent us a copy*, (don't know why the publishers did not; by their failure to do so they have unconsciously placed us under obligations) and wrote as follows:

"DEAR DR. DANIEL: For fear it would not fall under your notice, I send you Vol. 1. No. 1 of *Moody's Medical Magazine*.

It is edited, I see, by a young man, (yes, I judge he is very young) just out of college. I had hoped that Southern medical journalism would not ape this Yankee idea of filling a medical journal with such stuff as you will find in this one. I see it is full of the productions of the editor, whose name appears to several articles, and on the title and editorial pages, varying all the way from plain R. H. Bell to *Ralcey Husted* Bell, etc.

"Yours truly,

"——————, M. D."

Hear the bazoo of this Bell! *Ralcey* Bell! What a world of stuff he *does* give us! Well! How he swells! How he dwells upon each "cure!" (What a *cacoethese scribendi* he has, to be sure!) It's Bell! Bell! Bell! all through. Copious Bell! How he tells us all he knows, and more, too! Oh, who can read the pretty rhyming screed, descriptive of the picture on that page,—read and not deplore the writers gone before, and deprecate th' degenerated age! Prurient Bell! (He's young, I guess, and green. Let's hope, that with age and antiseptic soap, he'll be clean). [With apologies to Poe].

Then, there's another innovation, "Department of Gynecology and Obstetrics: Edited by Virgil O. Hardon, M. D.," and if you don't believe it, here's Hardon himself, his picture, on the cover; a nice looking young fellow with his hair parted in the middle. We don't want to be Hard-on the young man, but it is *so* like Squeers, the Yorkshire schoolmaster, and Smike.

"Smike, spell horse."

"H-o-r-s-e—horse," says Smike.

"Correct: go and curry my horse so you won't forget it," says Squeers.

